# 

**Published:** 1847-02

**Authors:** 


### CONTENTS

OF NO. II.

---

### ORIGINAL COMMUNICATIONS.

### ESSAYS AND CASES.

Art. I.—Notes on Medical Matters and Medical Men in Paris. By David W. Yandell, M. D., of Louisville, Ky. - - - - - - - - 93

Art. II.—A Case of Compound Fracture of the Skull; with depression of both tables, and laceration of the Membranes and Substance of the Brain. By T. B. Greenley, M.D., of Westpoint, Ky. - - - - 113

Art. III.—Notes on Epidemic Scarlet Fever and Measles. By Silas Ames, M.D., of Montgomery, Ala. - 118

Art. IV.—Remarks on the Pathology and Treatment of Fever. By James C. Harris, M.D., of Wetumpka, Alabama. - - - - - - - - - 128

Art. V.—A Notice of the Grayson Springs. By Lunsford P. Yandell, M.D. - - - - - - 142

### BIBLIOGRAPHICAL NOTICES.

Art. VI.—Thoughts on the Effects of Age on the Human Constitution. A Special Introductory. By Chas. Caldwell, M. D., &c. Lecture on Obstetrics and the Diseases of Women and Children. By G. S. Bedford, M.D., &c. - - - - - - 144

### SELECTIONS FROM AMERICAN AND FOREIGN JOURNALS.

Division of the Stricture in Hernia. - - - - - - 153

On the Digestion of Alcoholic Liquids and their Operation in Nutrition; with Hygienic Applications. - - - 154

Alkalies in the Treatment of Hooping-Cough. - - - 155
Artificial Somnambulism in Pennsylvania. - - - 156
Obliteration of the Œsophagus. - - - - - - 158
Method of Operating in Excision of the Cervix Uteri. - 160
Mortality from Cancer in England, as regulated by Sex. - 163
Electricity in Disease. - - - - - - - - 163
Advance of the Asiatic Cholera towards Europe. - - 166
Black Oxide of Mercury in the Vomiting of Pregnancy. - 167
Mortality in the Army and Navy. - - - - - 168
Operation for Aneurism. - - - - - - - 169
Hydrate of Lime in Diarrhœa. - - - - - - 170
Dr. Wollaston, the Chemist. - - - - - - 170
Ttreatment of White Swelling. - - - - - 172
Organic Chemistry and Medicine. - - - - - 173
Experimental Researches on the Nutritive Power of Green and Dry Fodder. - - - - - - - - 174
Notices of the Progress of Experiments on the Influence of Light on the Growth of Plants. - - - - - 175

ORIGINAL INTELLIGENCE.

Medical Department of the University of Louisville. - - 176
Other Western Schools. - - - - - - - 178
Ohio Lunatic Asylum. - - - - - - - 178
Professor Dickson's Introductory Lecture. - - - 181

